# Automatic personal identification using a single CT image

**DOI:** 10.1007/s00330-024-11013-x

**Published:** 2024-08-22

**Authors:** Andreas Heinrich

**Affiliations:** Department of Radiology, Jena University Hospital—Friedrich Schiller University, Jena, Germany

**Keywords:** Computer vision systems, X-ray computed tomography, Emergency care, Maxillary sinus, Human identification

## Abstract

**Objectives:**

Computer vision (CV) mimics human vision, enabling computers to automatically compare radiological images from recent examinations with a large image database for unique identification, crucial in emergency scenarios involving unknown patients or deceased individuals. This study aims to extend a CV-based personal identification method from orthopantomograms (OPGs) to computed tomography (CT) examinations using single CT slices.

**Methods:**

The study analyzed 819 cranial computed tomography (CCT) examinations from 722 individuals, focusing on single CT slices from six anatomical regions to explore their potential for CV-based personal identification in 69 procedures. CV automatically identifies and describes interesting features in images, which can be recognized in a reference image and then designated as matching points. In this study, the number of matching points was used as an indicator for identification.

**Results:**

Across six different regions, identification rates ranged from 41/69 (59%) to 69/69 (100%) across over 700 possible identities. Comparison of images from the same individual achieved higher matching points, averaging 6.32 ± 0.52% (100% represents the maximum possible matching points), while images of different individuals averaged 0.94 ± 0.15%. Reliable matching points are found in the teeth, maxilla, cervical spine, skull bones, and paranasal sinuses, with the maxillary sinuses and ethmoidal cells being particularly suitable for identification due to their abundant matching points.

**Conclusion:**

Unambiguous identification of individuals based on a single CT slice is achievable, with maxillary sinus CT slices showing the highest identification rates. However, metal artifacts, especially from dental prosthetics, and various head positions can hinder identification.

**Clinical relevance statement:**

Radiology possesses a multitude of reference images for a CV database, facilitating automated CV-based personal identification in emergency examinations or cases involving unknown deceased individuals. This enhances patient care and communication with relatives by granting access to medical history.

**Key Points:**

- *Unknown individuals in radiology or forensics pose challenges, addressed through automatic CV-based identification methods*.
- *A single CT slice highlighting the maxillary sinuses is particularly effective for personal identification*.
- *Radiology plays a pivotal role in automated personal identification by leveraging its extensive image database*.

## Introduction

Unknown individuals pose a significant challenge in emergency medicine and forensic investigations. Trauma centers frequently encounter unknown patients due to various circumstances such as natural disasters, terrorist attacks, severe accidents, migration, or homelessness [1–6]. Similarly, identifying unknown deceased individuals is difficult when there are no restrictive clues available about their identity. Computed tomography (CT) is beneficial in these scenarios, aiding diagnosis and personalized treatment in emergency care, and serving as a valuable tool for virtual autopsies in forensic investigations [7–10].

A novel computer vision (CV)-based method [11–13] has been introduced for automatic personal identification. This involves extracting distinctive CV features from images, such as orthopantomograms (OPGs), and storing them in an antemortem CV database. Identification is highly probable when there is a strong similarity between the CV features of an image of an unknown individual and a reference image.

Previous studies [14–18] demonstrated the utility of paranasal sinuses for accurate personal identification from CT slices. However, these methods require expertise, involving segmentations and training convolutional neural networks (CNNs) in some cases. The application of a CV can simplify and automate the personal identification process. This study aims to extend the CV-based personal identification method from OPGs to CT examinations using single CT slices.

## Materials and methods

The study was approved by the local Institutional Review Board (IRB) at Jena University Hospital (registration number 2019-1505-MV). Due to the retrospective nature of the investigation, written informed consent was waived by the IRB.

### CT slice selection

**Fig. 1:**
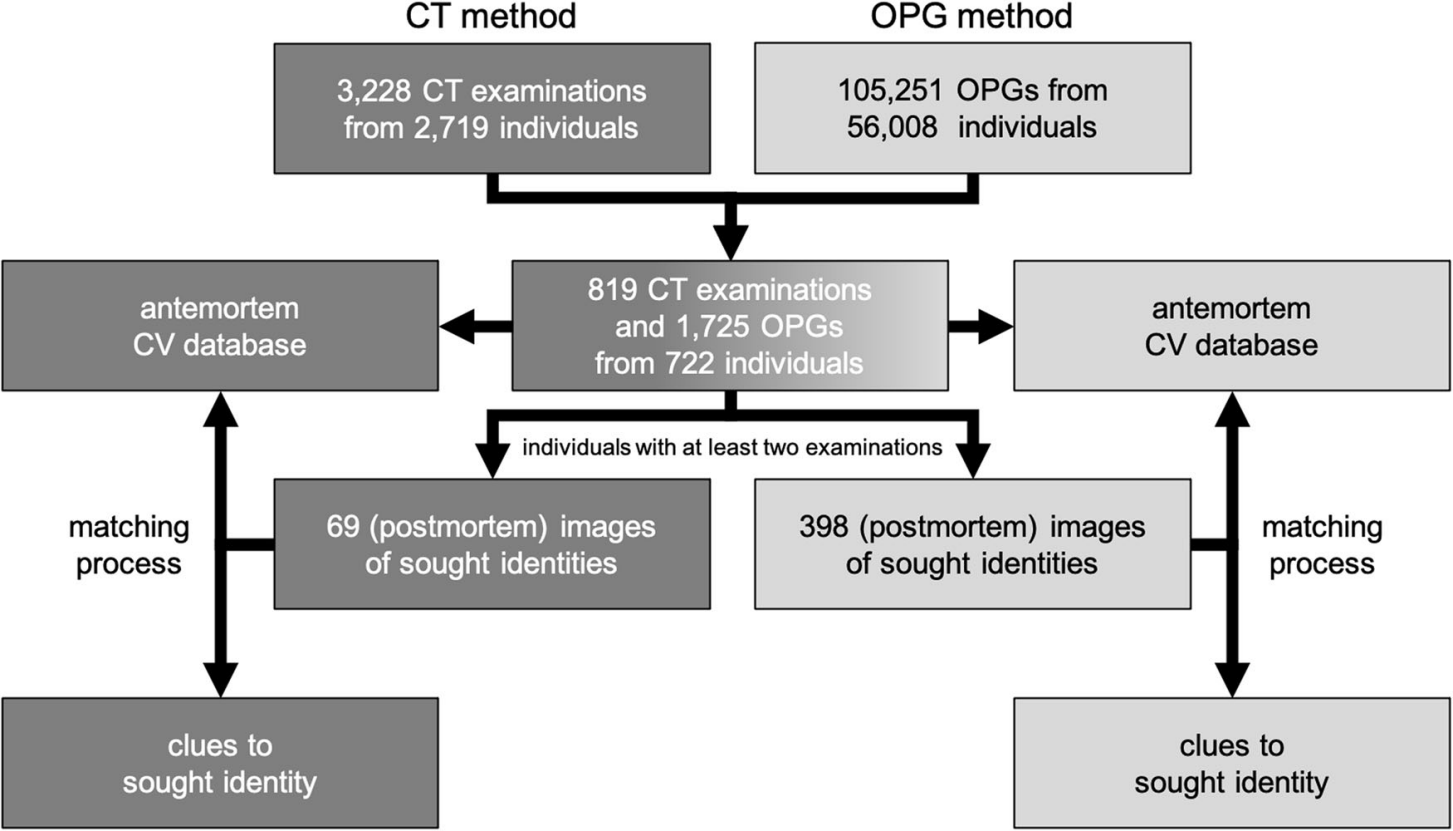
For a CCT protocol of the midface, only individuals with at least one OPG were selected. These examinations were further used to establish separate antemortem CV databases for the CT images and OPGs. Individuals with at least two examinations were used to match the most recent examination with the CV database and obtain clues to the identity of the result. If the result with the highest score (most matching points = best result) corresponds to the sought individual, then the identification is considered successful. Additionally, it’s possible to analyze, for example, the top ten results

The retrospective study examined 819 cranial computed tomography (CCT) examinations performed between November 2016 and May 2023 using a GE Revolution scanner (voltage: 120 kVp, current: 89.39 ± 21.21 mA, and slice thickness: 2.50 mm). It included 722 individuals (ages 10–99 years, mean age: 64.28 ± 21.27 years; 279 females, 402 males, and 41 unspecified). The inclusion criteria required a specific midface visualization protocol and at least one corresponding OPG for each individual. Figure 1 illustrates the selection process, which identified 819 CT examinations and 1725 OPGs (acquired between December 2000 and May 2023) meeting the criteria out of 3228 CT examinations and 105,251 OPGs reviewed.

Six regions were defined, and their CT slices were manually selected:

- Lower row of teeth.
- Upper row of teeth.
- End of maxilla.
- Cervical spine.
- Maximal representation of maxillary sinuses.
- Maximal representation of eye structures.

**Fig. 2:**
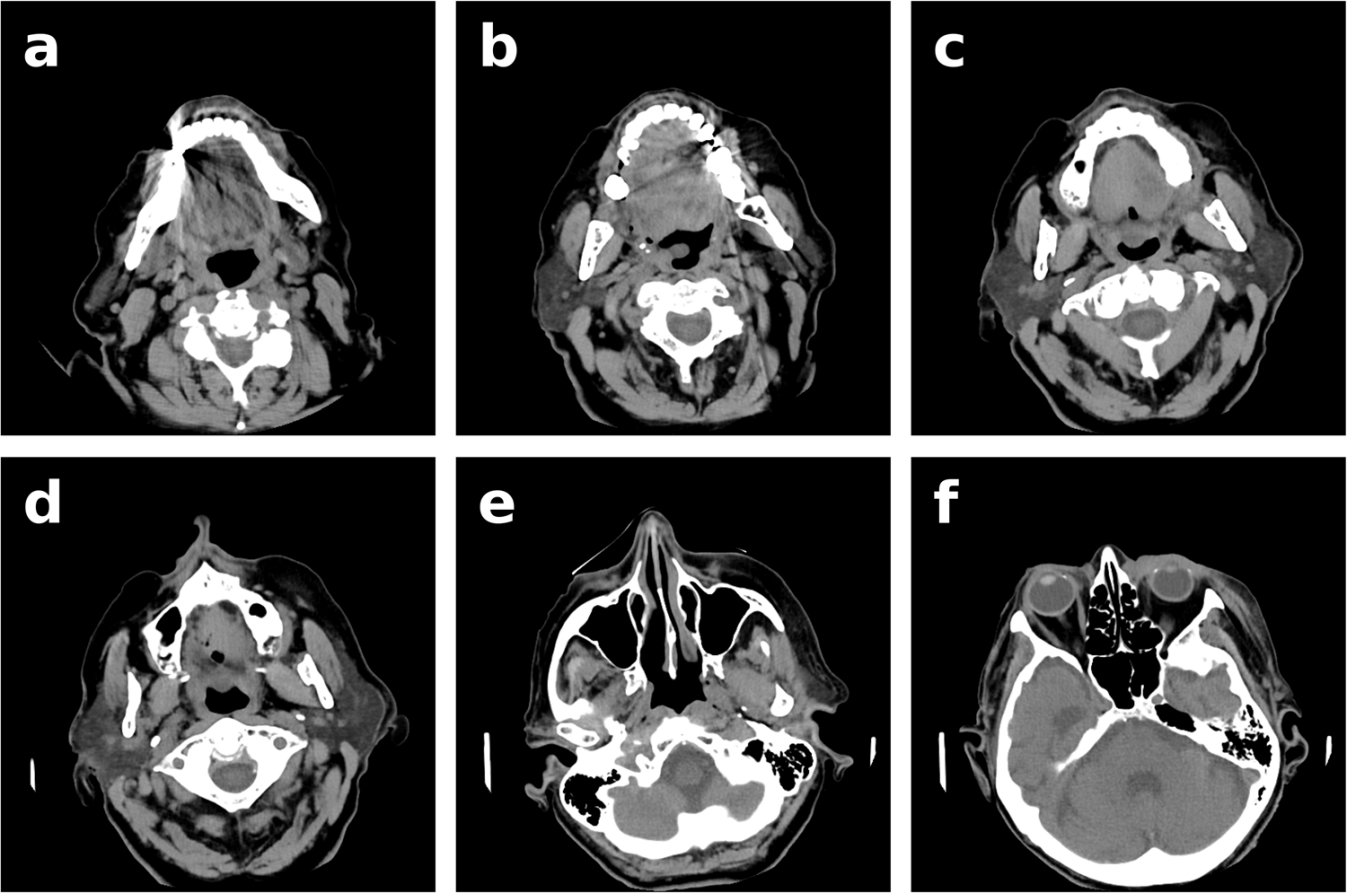
CT single slices (regions) used in the study: (**a**) lower row of teeth, (**b**) upper row of teeth, (**c**) end of maxilla, (**d**) cervical spine, (**e**) maximal representation of maxillary sinuses, and (**f**) maximal representation of eye structures

Figure 2 provides examples of CT slices for a–f. Missing teeth, large metal artifacts (caused, for example, by dental implants), or other artifacts (e.g., movement during the examination) hindered the location of all categories, preventing their use in the subsequent identification process.

### CV feature extraction and matching process

The image processing and identification methods were adapted from previous studies on OPGs [11–13]. Unlike OPGs, no cropping of the edges of the CT slices was performed [11]. The CT slices underwent several processing steps, including color depth normalization to 8 bits, edge highlighting using eight modified Sobel filter masks, noise reduction with an averaging filter, and feature extraction using the CV algorithm AKAZE [19]. CV automatically identifies and describes robust image features such as corners, edges, and other distinctive details, resilient against rotation, scale, and lighting changes. A CV feature consists of a keypoint and a descriptor. Keypoints mark distinctive points in the image, while descriptors provide a compact visual representation of the keypoint’s surroundings. The AKAZE algorithm utilizes octaves to generate a series of scaled-down images from the original, applying nonlinear diffusion filtering within each octave to produce multiple layers with varying levels of smoothing. This approach aids in detecting features across different scales of detail. AKAZE identifies potential keypoints by detecting scale-space extrema across these filtered images. For keypoints to be confirmed, they must exceed a contrast threshold relative to their surroundings. Each keypoint is then oriented based on local information and characterized by intensity and gradient details in its descriptor. This phase comprises seven parameters influencing the number and type of exported CV features:

- Sobel gradient: to enhance edges in CT slices, modified Sobel filters were utilized at various angles ranging from 0° to 315° in 45° increments. For example, at 0°, the matrix (− a 0 a, − 2a 0 2a, − a 0 a) highlights horizontal edges from left to right, whereas at 90°, the matrix (− a − 2a − a, 0 0 0, a 2a a) emphasizes vertical edges from top to bottom. The intensity adjustment was performed using a parameter referred to as Sobel gradient (a), with a traditional Sobel gradient set at *a* = 1. By combining the maximum pixel values from all eight Sobel gradient images, an image highlighting edges was generated. Refer to Fig. S1 in the Supplementary Material for an illustration.
- Averaging filter: size of the kernel of the average filter to reduce image noise.
- Octaves: determines how many times the image is scaled down to detect features at different sizes. More octaves mean the algorithm can find both larger and smaller features.
- Layers: indicates the number of levels of detail examined within each octave. More layers mean finer details can be detected in the image.
- Diffusivity: determines how the algorithm processes edges and textures when creating feature descriptors (PM_G1: moderate contrast handling, PM_G2: stronger contrast effect, WEICKERT: gentle contrast impact and preserving details, CHARBONNIER: nonlinear response and better edge preservation).
- Descriptor type: defines the type of descriptors (KAZE: excellent for extracting features from objects seen from different angles, MLDB: efficiently represents local features using binary patterns, upright descriptors: these are not rotation-invariant).
- Threshold: determines which potential keypoints are selected as keypoints based on their contrast compared to the surrounding areas. Higher thresholds make the detection more selective, finding fewer but more distinct features.

The detected CV features in the image, along with the study date, birth date, sex, and patient ID from the digital imaging and communications in the medicine header, were stored in an encrypted antemortem CV database.

**Fig. 3:**
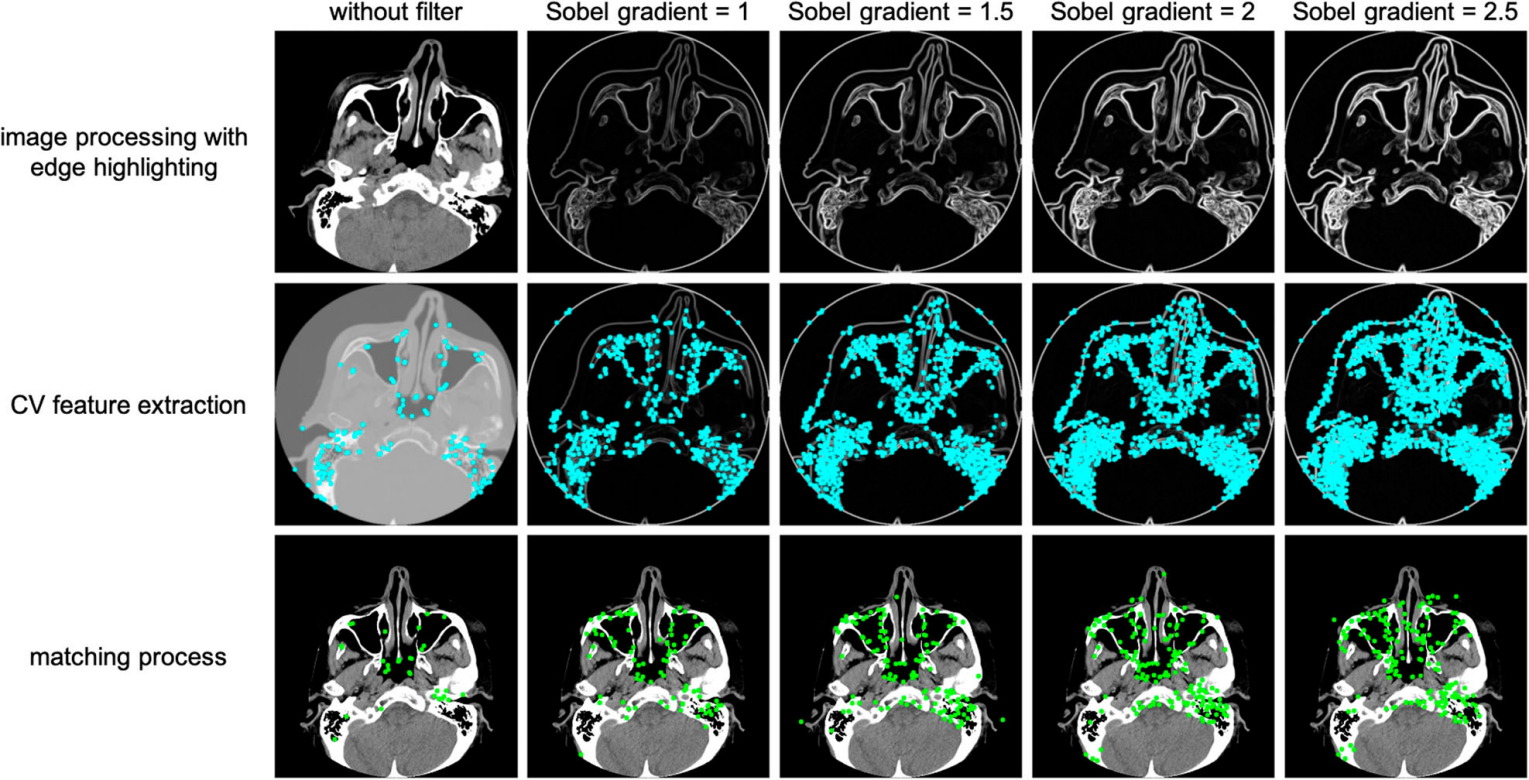
The figure illustrates processing steps in CV-based personal identification for different Sobel gradients, as well as without edge highlighting. Increasing the Sobel gradient allows for more CV features to be extracted (second row with blue dots). In the matching process, these CV features can be recognized in a reference image (third row with green dots). However, the Sobel gradient should not be too large, as this can lead to more noise and thus fewer distinctive matching points. For this reason, the systematic variation was only carried out up to a value of 2.5

During the matching process, the software compares CV features from an image of an unknown individual to CV features of reference images (entries in the database), with the number of matching points obtained serving as an indicator for identification. For each CV feature descriptor, the software identifies the two most similar descriptors in the reference image based on their smallest squared Euclidean distance. Subsequently, it applies Lowe’s test to ensure that only reliable matches are retained, thereby ensuring that the distance to the closest neighbor (most similar descriptor) is significantly smaller than to the second-closest neighbor. Each CV feature has at most one corresponding match to ensure unique matching points. The random sample consensus (RANSAC) algorithm is then used to further enhance accuracy by eliminating outlier matching points. RANSAC tests various scenarios to determine the optimal model fit, ensuring that misleading matching points are excluded. Figure 3 shows an example of edge highlighting using different filter settings, followed by CV feature extraction and the resulting matching points when compared with another image. The matching process involved two parameters, affecting the final number of matching points:

- Lowe: sets the threshold ratio for Lowe’s test. For example, with a threshold ratio of 0.5, the best match must be at least twice as close as the second-best match.
- RANSAC: determines the threshold to distinguish inliers and outliers. It uses an iterative approach to estimate model parameters from observed data points, ensuring accuracy by removing outlier matching points.

The number of remaining matching points can be normalized with the number of CV features in the search image, resulting in a score between 0% (no match) and 100% (identical images), describing the degree of similarity between two images:

$${{{\rm{Score}}}}={{{{\rm{Matching}}}}\; {{{\rm{points}}}}}/{{{{\rm{Total}}}}\; {{{\rm{CV}}}}\; {{{\rm{features}}}}}\, [ \% ]$$

For example, if the image of the unknown individual has 1000 CV features and the matching process yielded 50 matching points, then the score is 5%. In this study, matching was performed twice, first with the order “search image–database entry” and then with the order “database entry–search image”. The score was calculated as the average of matching points from these two runs.

### Parameter optimization and evaluation

To determine optimal parameters a systematic parameter optimization was conducted for each region. Starting with the parameter setting column “start value” in Table 1, the Sobel gradient was adjusted from 1.2 to 2.5. Then, the averaging filter parameter varied from 0 to 10 based on the optimal Sobel gradient. Subsequently, the octaves parameter was adjusted from 3 to 10 using the optimal values for the Sobel gradient and averaging filter. This process continued until all parameters were determined. The optimal setting was chosen based on the highest identification rate. If multiple parameters yielded the same rate, various approaches were tested to find the best one. The modal value of the optimal parameters from all regions was applied to achieve a universal parameter setting. For OPGs, parameters previously determined as optimal through systematic variation [13, 20] were adopted (Sobel gradient 1.8, averaging filter 6, octaves 8, layers 5, diffusivity PM_G2, descriptor type KAZE_UPRIGHT, Lowe 0.6, and RANSAC 10).

**Table 1:** The parameters were systematically varied to determine the optimal settings for each region a–f

Variable	Variation, [step size]	Start value	Optimal settings after systematic variations						
			a	b	c	d	e	f	Mode
Sobel gradient	1.2–2.5 [0.1]	–	1.4	2.4	1.8	2.3	1.8	2.4	1.8
Averaging filter	0–10 [1]	3	3	3	3	5	3	3	3
Octaves/layers	3–10 [1]	4/4	4/4	7/4	4/4	5/4	4/4	4/4	4/4
Diffusivity	1 = PM_G1 2 = PM_G2 3 = WEICKERT 4 = CHAR-BONNIER	2	2	2	2	2	2	4	2
Descriptor type	1 = MLDB 2 = MLDB upright 3 = KAZE 4 = KAZE upright	1	3	3	3	3	3	1	3
Threshold	0.0005–0.0025 [0.0005]	0.001	0.001	0.002	0.001	0.001	0.001	0.001	0.001
Lowe	0.1–1 [0.1]	0.6	0.7	0.6	0.6	0.6	0.5	0.7	0.6
RANSAC	1–15 [1]	10	3	2	2	2	2	2	2

Subsequently, the modal values (mode) of the optimal parameters were utilized to establish uniform parameter settings for each

For unique personal identification, between 50 and 69 CT slices per region (the most recent ones from individuals with at least two examinations) were compared with up to 819 CV database entries. In Table 2, the individual values for each region are listed. Additionally, 398 OPGs were matched with 1725 OPGs of the same individuals. Further optimization of the identification method occurred by replacing the manually extracted search image from the CT series with up to three slices before and after it. This means that not only the initially selected slice but also the adjacent slices were included in the search process. From the seven search images per identification case, the result with the highest score was then chosen for the subsequent outcomes.

**Table 2:** For 69 identification procedures, 50–69 CT slices (column “images for matching”) were matched with the CV database containing between 652 and 819 entries from 579 to 722 individuals

Region	Images for matching	CV database		Reference images per sought identity	
		Entries	Individuals	Mean value	Median
a	54	698	619	1.39 ± 0.74	1.00
b	50	652	579	1.38 ± 0.73	1.00
c	69	819	722	1.35 ± 0.69	1.00
d	68	806	712	1.34 ± 0.69	1.00
e	69	815	719	1.35 ± 0.69	1.00
f	69	815	718	1.35 ± 0.69	1.00
OPG	398	1725	722	2.53 ± 2.27	2.00

As a reference with OPGs (region OPG), 398 identification procedures were compared with 1725 database entries. Additionally, the mean and median of the reference images per sought identity available in the CV database for identification were provided

The identification rate assesses the accuracy of recognizing individuals. “Rank 1” means the score for images of the same identity surpasses scores for all other identities. In “rank 5” and “rank 10”, the score for the sought individual ranks within the top 5 or 10 scores among all scores per identity. Statistical significance (α = 0.05) was assessed using the Mann–Whitney *U-*test to compare score distributions between groups (same identity vs different identities), implemented with Python’s SciPy library.

## Results

In this study, a total of 819 CCT examinations of 722 individuals were used to evaluate whether a unique identification via single CT images is possible in 69 identification procedures. For six different regions, an identification rate ranging from 59% to 97% for rank 1 and 67% to 100% for rank 10 could be achieved (refer to Table 3 results with modal parameters and best search image). Region e was highly successful, accurately identifying the sought individuals in 67 out of 69 cases (97%) at rank 1. In the remaining two cases, both the first and second-ranked individuals had identical scores, with the sought individuals included in both instances.

**Table 3:** Results of CV-based personal identification for six CT slice regions a–f (refer to Fig. 2) and OPGs as reference

Region	Score, [%]				DIFF MED	Identification rate, [%]		
	MW, (=)	MED, (=)	MW, (≠)	MED, (≠)		Rank 1	Rank 5	Rank 10
Results after systematic parameter variations								
a	7.40 ± 7.50	4.99	1.84 ± 0.68	1.71	3.28	49.28	55.07	60.87
b	4.63 ± 3.67	3.55	0.86 ± 0.61	0.86	2.69	56.52	60.87	62.32
c	4.20 ± 3.79	3.12	0.94 ± 0.41	0.88	2.24	69.57	76.81	78.26
d	4.58 ± 4.62	3.50	0.91 ± 0.46	0.86	2.64	69.57	78.26	82.61
e	4.00 ± 4.64	2.41	0.34 ± 0.27	0.33	2.08	88.41	94.20	95.65
f	3.32 ± 4.35	1.93	0.68 ± 0.22	0.65	1.28	71.01	79.71	82.61
OPG	10.51 ± 12.45	6.58	0.30 ± 0.46	0.21	6.37	85.68	87.69	88.44
Results with modal parameters								
a	4.18 ± 4.91	2.63	1.08 ± 0.39	1.03	1.60	46.38	52.17	53.62
b	4.48 ± 3.43	3.45	1.03 ± 0.45	0.97	2.48	53.62	59.42	65.22
c	4.20 ± 3.79	3.12	0.94 ± 0.41	0.88	2.24	69.57	76.81	78.26
d	4.70 ± 5.14	3.45	0.99 ± 0.40	0.93	2.52	66.67	73.91	78.26
e	4.93 ± 5.15	3.04	0.65 ± 0.22	0.61	2.43	86.96	97.10	97.10
f	5.83 ± 6.78	3.48	1.02 ± 0.43	0.95	2.53	73.91	84.06	84.06
OPG	2.70 ± 5.58	1.28	0.25 ± 0.32	0.17	1.11	84.67	87.44	88.44
Results with modal parameters and the best search image								
a	5.81 ± 6.75	3.74	1.08 ± 0.40	1.03	2.71	59.42	62.32	66.67
b	5.99 ± 5.62	4.23	1.05 ± 0.45	1.00	3.23	63.77	68.12	68.12
c	6.17 ± 6.89	4.19	0.90 ± 0.38	0.85	3.34	79.71	89.86	91.30
d	5.88 ± 6.46	4.34	0.97 ± 0.38	0.91	3.43	82.61	88.41	92.75
e	6.96 ± 6.96	4.82	0.64 ± 0.23	0.60	4.22	97.10	100	100
f	7.13 ± 6.75	4.76	1.00 ± 0.42	0.94	3.82	95.65	98.55	98.55

MW denotes the mean value, and MED represents the median for the same individual (=) or different individuals (≠) of the score (number of matching points divided by the total number of CV features in the search image). DIFF MED is the difference in the median scores between the same individual and different individuals

**Fig. 4:**
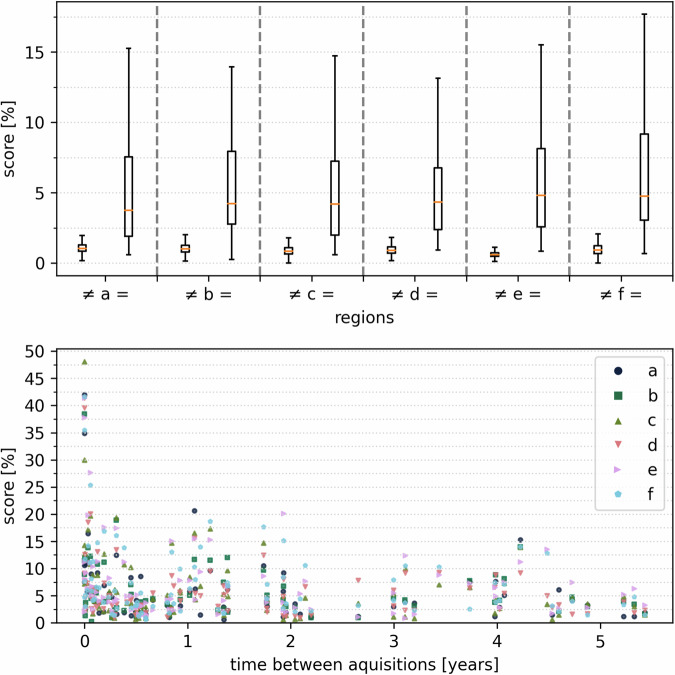
The boxplot (above) displays the results of CV-based personal identification for six CT slice regions a–f (refer to Fig. 2), illustrating comparisons between the same individual (=) and different individuals (≠). The score represents the number of matching points divided by the total number of CV features in the search image. The subfigure below illustrates the relationship between the score and the time between CT acquisitions of the same individual, demonstrating successful identification even for images taken several years apart. To ensure comparability of the scores for this figure

Comparison of images from the same individual achieved higher matching points, scoring 6.32 ± 0.52%, while images of different individuals scored 0.94 ± 0.15% (see Fig. 4). For all regions, the *p*-value was < 0.001, indicating a highly significant difference between the groups. Figure 4 shows the relationship between the score and the time between CT acquisitions of the same individual, demonstrating successful identification even for CT examinations taken several years apart. The identification rate and thus the score depends on the parameter settings used (refer to Supplementary Material Fig. S2).

**Fig. 5:**
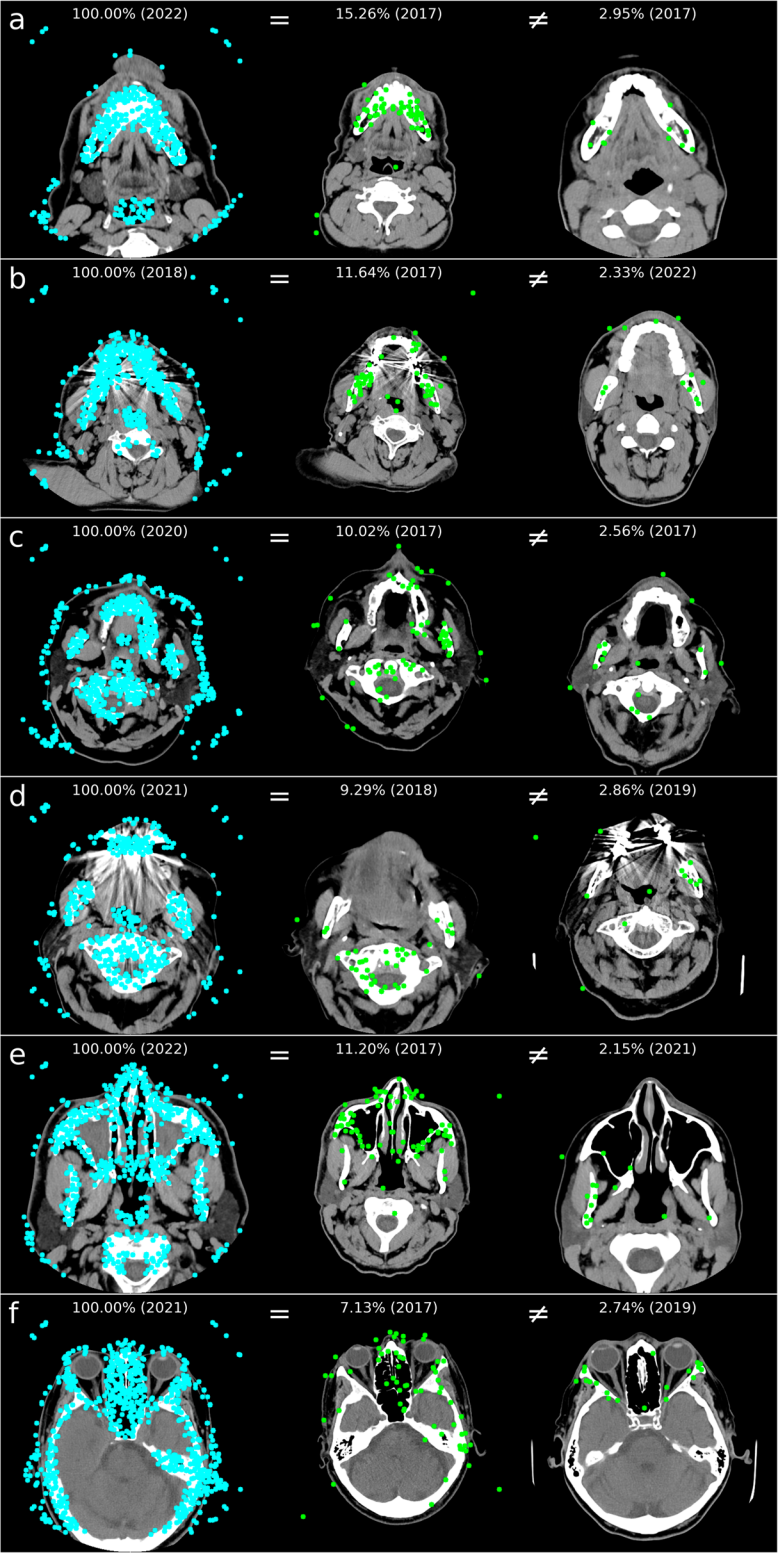
Examples of search images (left) for regions (**a**–**f**) and the best result (highest score) for the same identity (“=“, middle) and for different identities (“≠“, right) with the indication of the score in percentage and the image acquisition year in parentheses. Although structures may exhibit similarities leading to matching points, these similarities are typically much lower between different individuals compared to the same individual

In Fig. 5, an example of identified matching points is depicted. While teeth also exhibit many matching points (compared with Fig. 5a), metal artifacts disrupt the clear identification (compare with Fig. 5b). However, the skull bone, cervical spine, and cavities such as the maxillary sinuses and the ethmoidal cells provide many distinctive features that can be recognized (compare with Fig. 5c–f). For comparison, 1725 OPGs of the same individuals were used as a reference method for 398 identification procedures. Here, an identification rate of 86% (rank 1) to 88% (rank 10) could be achieved. The higher scores for different individuals observed in CT slices compared to OPGs, may be attributed to various factors including metal artifacts, lower image resolution, and the presence of similar objects such as the CT table, medical equipment, or limitations in the field of view within the image. Although structures may exhibit similarities leading to matching points, these similarities are typically much lower between different individuals compared to the same individual. The various head positions during imaging of the search image and the reference image can complicate the identification, as fewer matching points may be found.

**Fig. 6:**
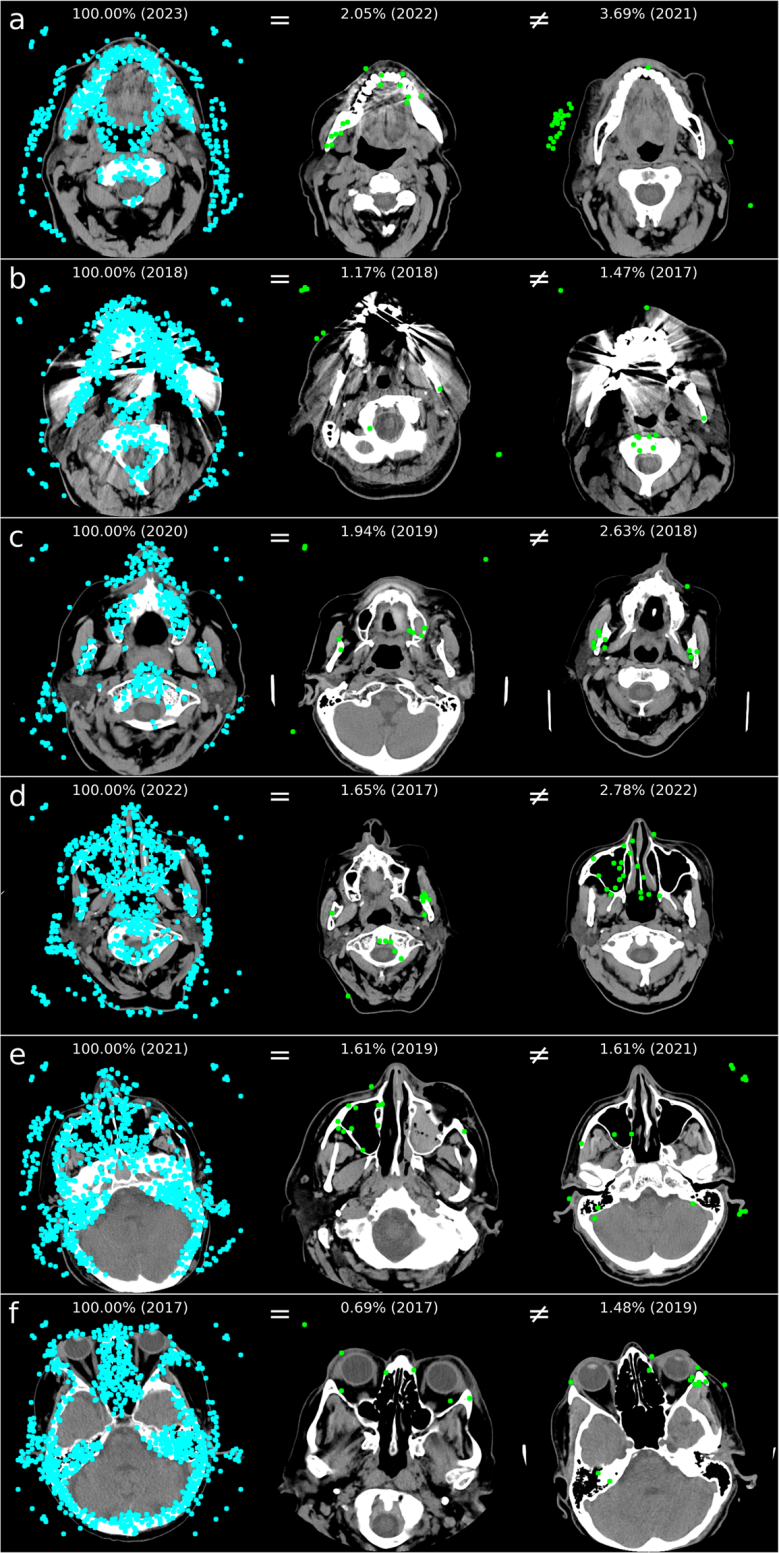
For unsuccessful identifications, examples of search images (left) for regions (**a**–**f**) and the best result (highest score) for the same identity (“=“, middle) and for different identities (“≠“, right) with the indication of the score in percentage and the image acquisition year in parentheses. Similar objects, like medical accessories (**a**) or CT tables, can result in a higher score for different individuals. Metal artifacts can also hinder identification (**b**). The various head positions during imaging of the search image and the reference image can complicate personal identification, as fewer matching points may be found (**c**–**f**)

For regions a and b, metal artifacts from dental implants and the absence of teeth are particularly decisive factors for unsuccessful identification. In contrast, for regions c–f, unfavorable head positions are more often reasons for unsuccessful identification (refer to Fig. 6 for examples). In Table 4, reasons were summarized for results in identification procedures that did not achieve at least rank 10.

**Table 4:** The table summarizes reasons for unsuccessful identification processes, even at rank 10

Reasons	a	b	c	d	e	f
Defined region not found in the search image or reference image, for example, due to missing teeth or large metal artifacts	15	19	0	1	0	0
Various head positions (search image and reference image not ideally comparable)	3	0	4	3	0	1
Metal artifacts in search image and/or reference image	2	3	0	1	0	0
Various head positions and metal artifacts	0	0	2	0	0	0
Medical equipment (resulting in more matching points for different identities)	3	0	0	0	0	0
Sum	23	22	6	5	0	1

The evaluation utilized the parameter setting that achieved the highest rank 10 in Table 3

## Discussion

This study demonstrates the feasibility of CV-based personal identification using a single CT slice of the head with an identification rate of up to 100% across 69 identification procedures and over 700 possible identities. In CV, distinctive areas exhibit more unique features, particularly at edges, corners, and intersections of lines [19, 21]. The CV features remain relatively consistent across rotation, scale, and lighting changes, which makes them excellent markers. Teeth, maxilla, cervical spine, skull bones, and cavities such as the maxillary sinuses and ethmoidal cells have numerous edges. However, metal artifacts pose a problem as they can obscure these edges, leading to poorer identification rates, especially by dentures in the lower and upper rows of teeth (regions a and b). Varying head positions introduce additional challenges, causing an inconsistent representation of anatomical features and consequently fewer matching points. Nevertheless, large cavities such as the maxillary sinuses (region e) have many matching points for the same identity, even across multiple CT slices, due to their consistent edges. Similarly, but to a slightly lesser extent, the maximal representation of eye structures (region f) benefits from the ethmoidal cells and large skull bones, which also show many matching points across multiple CT slices. Therefore, even with varying head positions, sufficient matching points within the defined regions are found, ensuring reliable identification. In contrast, other regions are more vulnerable to variations in head position due to potentially insufficient matching points within the defined region and inconsistent edges across multiple CT slices. The region-specific dependence of matching points on optimal search and reference images, also influenced by varying head positions, is illustrated in Supplementary Material Figs. S3 and S4.

To my knowledge, there is currently no existing research in the literature on the application of CV for unique identification based on single CCT slices. In previous studies [15–18], the forensic significance of paranasal sinuses, including the maxillary sinuses, in personal identification is highlighted. Their uniqueness is supported by substantial inter-individual variations in size, shape, symmetry, and outer contours, fulfilling criteria of uniqueness, permanence, and immutability. The high specificity of paranasal sinuses as a reliable area for personal identification is demonstrated, for instance, through visual examination of CT images by four blinded readers with varying levels of radiological experience [16], or by comparing self-measured parameters (morphometry) of the paranasal sinuses and evaluating these parameters using a CNN [17] or an iterative closest point algorithm based on segmented 3D images of the sphenoid sinus [18]. Nevertheless, employing a wholly different and automatable method, this study’s results confirm these findings. Similar to human assessment, the CV algorithm can identify and recognize numerous individual features. This research suggests that a single CT slice may suffice for this purpose, and the process can be automated, without the need for expert assessment or segmentation. Moreover, teeth and dental prosthetics are suitable for individual identification [22]; however, unlike in OPG, these can be obscured in CT examinations due to metal artifacts. On the other hand, CT examinations provide more accurate information about the maxillary sinuses compared to OPG [16, 23]. The optimal parameter selection is complex due to numerous possibilities. This study systematically varied parameters across different regions to find suitable settings, standardizing them beyond the modal values. Parameters affect the number of CV features and matching points, making scores comparable only under identical conditions. The goal is to maximize score differences between identical and different identities, thereby enhancing identification rates. For very similar images, such as those from re-examinations, high scores for the same individuals can lead to non-normal distributions in Fig. 4. Alternative edge detection filters like the Laplacian are viable, but the modified Sobel filter often achieves superior identification rates (see Supplementary Material Fig. S5). Using multiple search images across adjacent CT slices significantly improves the identification rate by reducing the impact of slight variations in the selection of search images and/or head positions. Another advantage of this approach is that the antemortem CV database remains unchanged.

The limitations include focusing solely on axial CT slices. Identification rates can be further improved by implementing filters based on sex, age, and acquisition date. Concealing disruptive elements in search images would prevent incorrect matches and improve reliability. Integrating complete CT series into CV databases would eliminate the need for manual selection of reference images based on defined regions. Future research could improve image comparability with multiplanar reformatting for accurate orientation and precise slices from thin-slice 3D datasets. The method could also benefit from reconstructing X-ray-like representations from CT data and comparing them with conventional X-rays and OPGs. This approach is particularly useful for young individuals undergoing their first CT examinations, enriching reference materials for accurate identification.

In conclusion, automated personal identification using CV features from single CT slices achieves high accuracy. Without employing additional measures like age and sex filtering, image segmentation, or multiplanar reformatting, the method successfully identified 97–100% of identities ranked 1–2 from a pool of over 700 possibilities. Radiology’s role in generating reference images during routine examinations for the CV database is crucial for rapid identification in emergencies and for unknown deceased individuals. This improves patient care by facilitating access to medical histories, assisting in determining the cause of death, and preserving potential crime scene evidence.

## Supplementary information

ELECTRONIC SUPPLEMENTARY MATERIAL

## Abbreviations

CCT: Cranial computed tomography
CT: Computed tomography
CV: Computer vision
OPG: Orthopantomogram
RANSAC: Random sample consensus
