# Supplementary material for: Automatic personal identification using a single CT image
Source: Eur Radiol. 2024 Aug 22;35(5):2422–33. doi: 10.1007/s00330-024-11013-x (PMC12021953; doi:10.1007/s00330-024-11013-x)
Supplement: Supplementary file 1 — ELECTRONIC SUPPLEMENTARY MATERIAL [file 330_2024_11013_MOESM1_ESM.pdf]

Automatic personal identification using a single CT image

ELECTRONIC SUPPLEMENTARY MATERIAL

Eur Radiol (2024) Heinrich A.

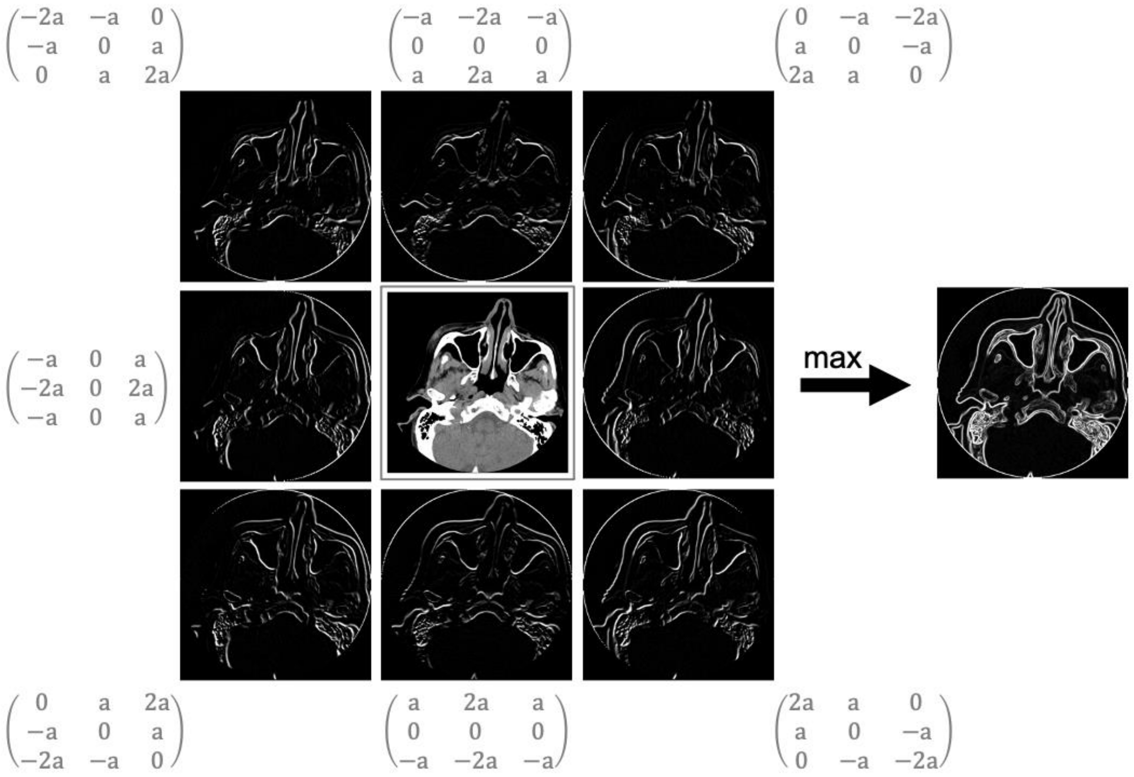

Figure S1 To perform edge highlighting, eight modified Sobel filter masks (ranging from 0° to 315° in 45° increments) were utilized to enhance the edges within the CT slices. The intensity of these edges was adjusted using a parameter called Sobel gradient (a), where a classic Sobel gradient is represented by a = 1. A unified image emphasizing edges in all directions was generated by aggregating the maximum pixel values from all eight Sobel gradient images.

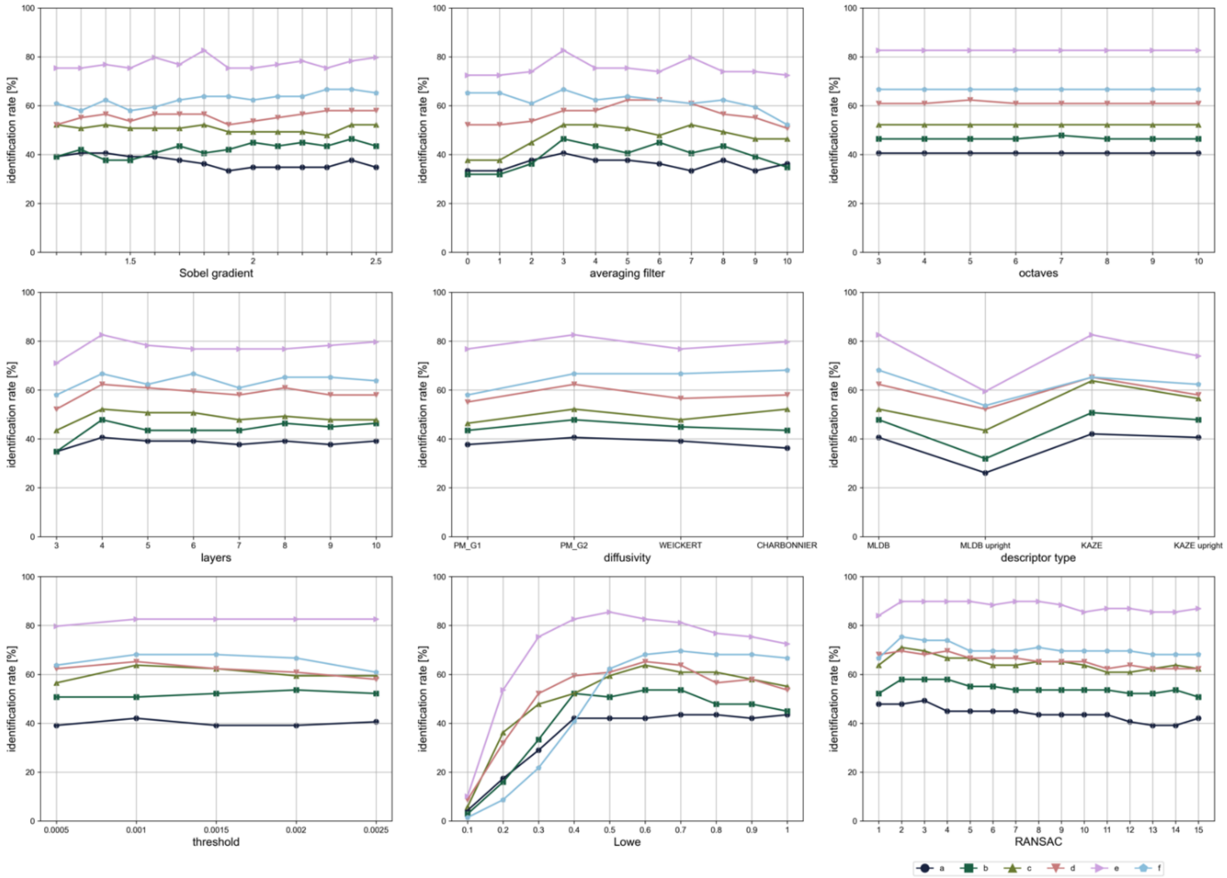

Figure S2 The identification rate for a systematic variation of parameters for six CT slice regions a-f (compare with Table 1). An average filter of zero means that this filter was not applied.

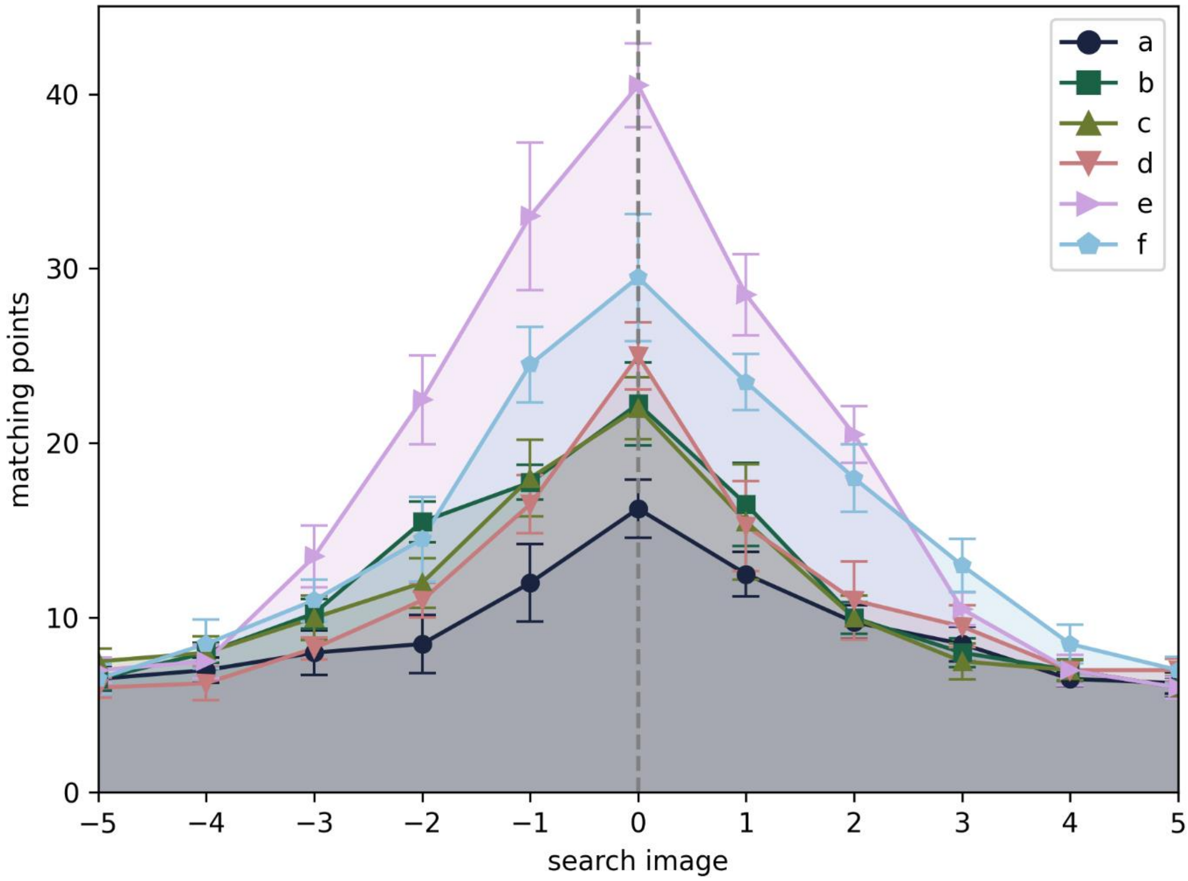

Figure S3 For a comparison of two images of the same identity across regions a-f, the median number of matching points with standard deviation is shown for 50-69 identification procedures. It becomes evident that the maxillary sinuses are less affected by various head positions or variations in the selection of the search image, as many matching points can still be found even with the shifting of the initial search image. This is attributed to the size of the cavities, which maintain consistent edges across multiple CT slices, allowing them to be reliably recognized and matched in CV. Also, skull bones and ethmoidal cells can exhibit comparable edges across multiple CT slices, which makes region f more reliable compared to regions a-d.

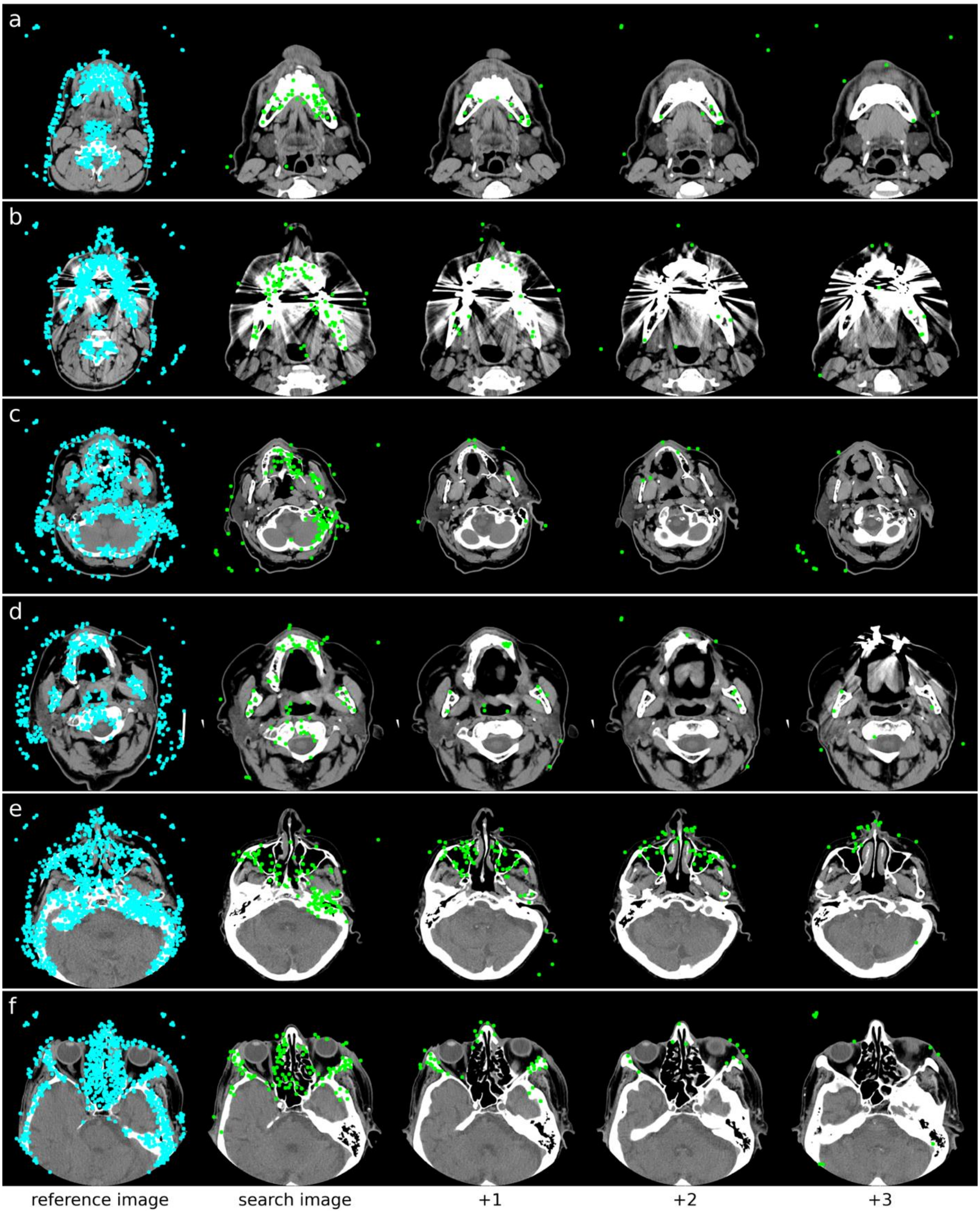

Figure S4 An example is shown of how the matching points decrease when shifting the initial search image by up to 3 slices. Inaccurate slice selection or various head positions between examinations can lead to deviations between the reference image (database entry) and the search image.

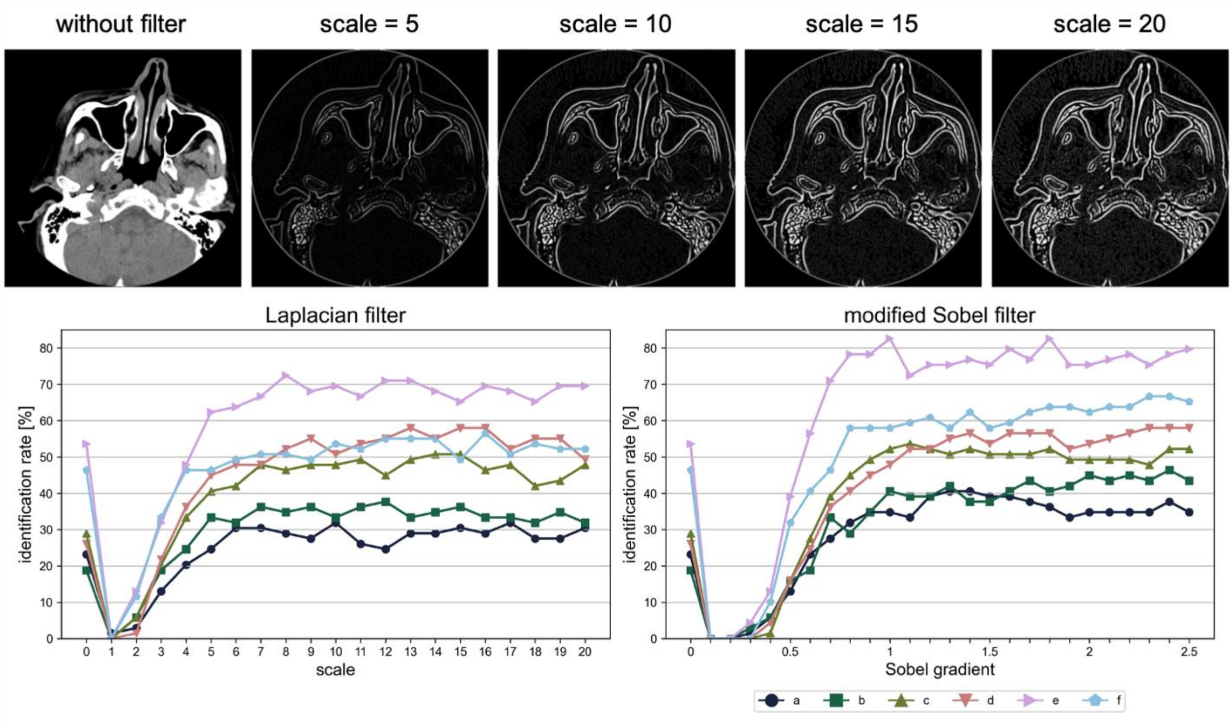

Figure S5 For edge highlighting, a Laplacian filter can be used instead of a modified Sobel filter. However, during the systematic variation of parameters, the modified Sobel filter often exhibited a higher identification rate, which is why it was used in this study (compare with Figure 3). A scale and Sobel gradient of zero means that these filters were not applied.
